# Endotoxin-induced inflammation down-regulates l-type amino acid transporter 1 (LAT1) expression at the blood–brain barrier of male rats and mice

**DOI:** 10.1186/s12987-015-0016-8

**Published:** 2015-09-04

**Authors:** Gábor Wittmann, Petra Mohácsik, Mumtaz Yaseen Balkhi, Balázs Gereben, Ronald M. Lechan

**Affiliations:** Division of Endocrinology, Diabetes and Metabolism, Department of Medicine, Tupper Research Institute, Tufts Medical Center, Boston, MA USA; Department of Endocrine Neurobiology, Institute of Experimental Medicine, Hungarian Academy of Sciences, Budapest, Hungary; Semmelweis University, János Szentágothai PhD School of Neurosciences, Budapest, Hungary; Division of Hematology/Oncology, Department of Medicine, Tupper Research Institute, Tufts Medical Center, Boston, MA USA; Department of Neuroscience, Tufts University School of Medicine, Boston, MA USA

**Keywords:** Inflammation, LPS, LAT1, Amino acid transport, Blood–brain barrier, Thyroid hormone

## Abstract

**Background:**

We recently reported that bacterial lipopolysaccharide (LPS)-induced inflammation decreases the expression of the primary thyroid hormone transporters at the blood–brain barrier, organic anion-transporting polypeptide 1c1 (OATP1c1) and monocarboxylate transporter 8 (MCT8). l-type amino acid transporters 1 and 2 (LAT1 & LAT2) are regarded as secondary thyroid hormone transporters, and are expressed in cells of the blood–brain or blood-cerebrospinal fluid barrier and by neurons. The purpose of this study was to examine the effect of LPS-induced inflammation on the expression of LAT1 and LAT2, as these may compensate for the downregulation of OATP1c1 and MCT8.

**Methods:**

LPS (2.5 mg/kg body weight) was injected intraperitoneally to adult, male, Sprague–Dawley rats and C57Bl/6 mice, which were euthanized 2, 4, 9, 24 or 48 h later. LAT1 and LAT2 mRNA expression were studied on forebrain sections using semiquantitative radioactive in situ hybridization. LAT1 protein levels in brain vessels were studied using LAT1 immunofluorescence. Statistical comparisons were made by the non-parametric Kruskal–Wallis and Dunn’s tests.

**Results:**

In both species, LAT1 mRNA decreased in brain blood vessels as soon as 2 h after LPS injection and was virtually undetectable at 4 h and 9 h. During recovery from endotoxemia, 48 h after LPS injection, LAT1 mRNA in brain vessels increased above control levels. A modest but significant decrease in LAT1 protein levels was detected in the brain vessels of mice at 24 h following LPS injection. LPS did not affect LAT1 and LAT2 mRNA expression in neurons and choroid plexus epithelial cells.

**Conclusions:**

The results demonstrate that LPS-induced inflammation rapidly decreases LAT1 mRNA expression at the blood–brain barrier in a very similar manner to primary thyroid hormone transporters, while changes in LAT1 protein level follow a slower kinetics. The data raise the possibility that inflammation may similarly down-regulate other blood–brain barrier transport systems at the transcriptional level. Future studies are required to examine this possibility and the potential pathophysiological consequences of inflammation-induced changes in blood–brain barrier transport functions.

**Electronic supplementary material:**

The online version of this article (doi:10.1186/s12987-015-0016-8) contains supplementary material, which is available to authorized users.

## Background

Circulating thyroid hormone (TH) enters the brain via the blood–brain and blood-cerebrospinal fluid barriers [1], by means of specific transporter proteins expressed in the membranes of cerebral endothelial cells and choroid plexus epithelial cells [2, 3]. Recent knockout mouse models provided evidence that TH entry into the brain is largely dependent on two TH transporters, monocarboxylate transporter 8 (MCT8) and organic anion-transporting polypeptide 1c1 (OATP1c1) [4–9]. We recently described that during systemic inflammation induced by bacterial lipopolysaccharide (LPS) administration, OATP1c1 and MCT8 mRNAs, as well as OATP1c1 protein markedly decrease in brain vessels [10]. The downregulation of these transporters at the blood–brain barrier would suggest reduced TH uptake into the brain and consequently, lower brain TH concentration, especially during prolonged systemic inflammatory states.

Other than MCT8 and OATP1c1, however, there are additional membrane proteins that are capable of transporting TH and may contribute to TH homeostasis in the brain [11, 12]. Included are type 1 and 2 L-type amino acid transporters (LAT1 or *Slc7a5*; LAT2 or *Slc7a8*) that transport TH with low to medium affinity [11, 13] and are expressed in multiple brain cell types. In vivo mouse and rat studies demonstrated that LAT1 is expressed in microvascular cells of the blood–brain barrier [12, 14–18], choroid plexus epithelial cells [14, 18] (in rats but not mice) and neurons [12], whereas LAT2 is expressed in neurons [12, 19, 20] and the choroid plexus [12, 19]. Therefore, LAT1 and LAT2 may contribute to TH transport across the blood–brain and blood-cerebrospinal fluid barriers and facilitate neuronal uptake and/or release of TH.

To determine whether upregulation of LAT1 and LAT2 compensates for the downregulation of MCT8 and OATP1c1 during inflammation and offsets the effect of reduced brain TH uptake on neurons, we examined the effect of LPS on LAT1 and LAT2 expression in the mouse and rat forebrain using in situ hybridization to study LAT1 and LAT2 mRNAs in a cell-type specific-manner, as well as immunofluorescence to study LAT1 protein levels.

## Methods

### Animals

The experiments were carried out on adult, male, Sprague–Dawley rats (Taconic Biosciences, Germantown, NY, USA) of similar age, weighing 220–260 g, and adult, male, C57Bl/6 mice (Taconic) of similar age, weighing 19–21 g. Animals were housed under standard conditions (lights on between 0600 and 1800 h, temperature 22 ± 1 °C, rodent chow and water ad libitum). Only male animals were used in this study to avoid the potential confounding effects of the estrous cycle on the experimental results. All experimental protocols were reviewed and approved by the Institutional Animal Care and Use Committee at Tufts Medical Center (Protocol# B2012-171) and was carried out in strict accordance with the recommendations in the Guide for the Care and Use of Laboratory Animals of the National Institutes of Health.

### Injections and tissue preparation

*LPS*-*injections* Animals were injected ip with LPS from *Escherichia coli* (serotype O55:B5; Sigma-Aldrich Co., St. Louis, MO, USA) between 9 and 11 a.m. LPS was dissolved in saline, and injected at a dose of 2.5 mg/kg body weight to all animals. Control animals received the same volume of saline. In our experience, different LPS serotypes such as O55:B55 and O127:B8 elicit very similar inflammatory responses in the brain, including upregulation of type 2 deiodinase [21, 22].

*Time course experiment for in situ hybridization in rats and mice* Groups of animals containing 4 or 5 rats or mice were injected with LPS, and 2, 4, 9, 24 or 48 h later were anesthetized with ketamine-xylazine (ketamine: 75 mg/kg body weight; xylazine: 10 mg/kg body weight), then decapitated. Control mice were euthanized at 9 h (n = 5), control rats at either 9 h (n = 3) or 24 h (n = 2), based on previous experiments. The intensity of hybridization and immunofluorescence signals were very similar in all control rats. The brains were removed, snap-frozen on powdered dry ice and 16 μm thick coronal sections from the forebrain were cut using a Leica CM3050 S cryostat (Leica Microsystems, Nussloch GmbH, Germany). Sections were thaw-mounted on Superfrost Plus slides (Fisher Scientific Co., Pittsburgh, PA, USA), air-dried and stored at −80 °C until processed for in situ hybridization or immunofluorescence.

### Generation of hybridization probes

Template cDNA fragments were generated with RT-PCR using standard procedures. Amplification was performed on cDNA synthesized from rat and mouse brain for LAT1 and rat and mouse liver for LAT2. Fragments were cloned into pGEM^®^-T vector (Promega) and confirmed by sequencing. Probe sequences were as follows: rat LAT1 probe corresponds to nt 64–851 of [GenBank: NM_017353.1]; mouse LAT1 probe nt 81–868 of [GenBank: NM_011404.3]; rat LAT2 probe nt 621–1368 of [GenBank: NM_053442.1]; mouse LAT2 probe nt 657–1335 of [GenBank: NM_016972.2]. Antisense and sense riboprobes were synthesized using SP6 or T7 RNA polymerase (Promega) in the presence of [35S]-uridine 5′-(alpha-thio) triphosphate (PerkinElmer), and purified with Mini Quick Spin RNA columns (Roche Applied Sciences).

### Isotopic in situ hybridization

Isotopic in situ hybridization was performed as previously described for fresh frozen sections [23] using 50,000 cpm/μl radiolabeled probe concentrations. Following stringency washes, sections were dehydrated in ascending ethanol series, air-dried and placed on Amersham Hyperfilm autoradiography film (GE Healthcare Biosciences) for 7 days. Slides were then dipped in Kodak NTB autoradiography emulsion (Carestream Health Inc) and stored at 4 °C until developed. Exposure times were as follows: 14 days for rat LAT1; 17 days for mouse LAT1; 14 days for rat LAT2 and 21 days for mouse LAT2. Autoradiograms were developed with Kodak D19 developer (Eastman Kodak Co). Sections were immersed in 0.0005 % cresyl violet acetate (Sigma-Aldrich) for 2 min to obtain fluorescent labeling of cell nuclei, dehydrated in ascending ethanol series and xylenes, and coverslipped with DPX (Sigma-Aldrich). Hybridizations with the sense transcripts for each probe did not yield any signal above a low-level of homogenous background. Darkfield images of the emulsion autoradiographs were captured using a Zeiss Axioplan 2 microscope equipped with a SPOT Slider digital camera (Diagnostic Instruments).

### Isotopic LAT1 in situ hybridization combined with immunofluorescence

To confirm that LAT1 mRNA is expressed in both microvascular cells and neurons, sections from control rat and mouse brains were hybridized for LAT1 as above, then processed further for immunofluorescence. The sections were treated with the mixture of 0.5 % Triton X-100 and 0.5 % H_2_O_2_ for 15 min, rinsed in phosphate-buffered saline (PBS) (3 × 10 min), immersed in maleate buffer (pH 7.5) for 10 min and then in 1 % blocking reagent for nucleic acid hybridization (Roche). To visualize neuronal cell bodies and blood vessels, sections were incubated in the mixture of a mouse monoclonal antibody against the neuronal proteins HuC/HuD (Life Technologies; Cat# A21271, raised against human HuC/HuD; used at 1 µg/ml) and polyclonal chicken IgY against vimentin (Millipore; Cat# AB5733, raised against recombinant vimentin; used at 1:4000 dilution). Other sections were incubated in a rabbit polyclonal antibody against LAT1 (Cosmo Bio Co., Ltd., Tokyo, Japan; Cat# KAL-KE026, diluted at 1 µg/ml) to label microvascular cells. The primary antibodies were detected with Alexa Fluor 488-conjugated donkey anti-mouse IgG (Life Technologies; 1:200), Cy3-conjugated donkey anti-chicken IgG (Jackson ImmunoResearch, West Grove, PA, USA) and Cy3-conjugated donkey anti-rabbit IgG (Jackson; 1:200). Sections were then dehydrated, dipped in Kodak NTB autoradiography emulsion, and the autoradiograms were developed after 17 days. The mouse HuC/D antibody and the chicken vimentin antibody have been used extensively for immunofluorescent studies (see references [22, 24–26]). The HuC/D antiserum labeled neuronal cell bodies, vimentin immunostaining labeled pericytes in the brain vasculature [27] as well as ependymal and meningeal cells.

### Immunofluorescence

For LAT1 immunofluorescence, fresh-frozen mounted sections were fixed with methanol at −20 °C for 5 min. While LAT1 immunostaining also worked well with standard 4 % paraformaldehyde fixation, methanol fixation produced more intense signal and less background staining. Sections were permeabilized with 0.25 % Triton-X-100 for 20 min, and then blocked with 2 % normal horse serum in PBS for 20 min. Sections were incubated overnight in a rabbit antiserum against human LAT1 (Cosmo Bio Co., Ltd., Cat# KAL-KE026, diluted at 1 µg/ml), which was subsequently detected with Cy3-conjugated donkey anti-rabbit IgG (Jackson Immunoresearch, 1:200).

To demonstrate that LAT1 is expressed in endothelial cells, dual immunfluorescence was performed for LAT1 and either CD31 (or PECAM-1) or glucose transporter 1 (GLUT1), both regarded as markers for endothelial cells. To label CD31, a monoclonal rat antibody against CD31 (Cat# 550274; clone MEC 1.3; BD Biosciences, Franklin Lakes, NJ, USA) was used at 0.3 µg/ml concentration and subsequently detected with Alexa Fluor 488-conjugated donkey anti-rat IgG (Jackson; 1:400). For GLUT1, a monoclonal mouse antibody against GLUT1 (Cat# MABS132; clone 5B12.3; Millipore) was used at 0.5 µg/ml concentration and subsequently detected with Alexa Fluor 488-conjugated donkey anti-mouse IgG (Life Technologies; 1:400).

LAT2 immunofluorescence was performed according to the same methanol fixation protocol as above using a rabbit polyclonal antiserum against mouse LAT2 (Cat# 0142-10; ImmunoGlobe Antikörpertechnik GmBH, Himmelstadt, Germany; diluted at 2 µg/ml), and subsequently Cy3-conjugated donkey anti-rabbit IgG (Jackson). This antiserum was used previously for immunohistochemistry in the mouse brain [20].

### Validation of the specificity of the LAT1 antiserum

The LAT1 polyclonal antiserum (Cosmo-Bio Co., Cat# KAL-KE026) was raised against the C-terminal 14 amino acids of human LAT1 (amino acid sequence: CTVLCQKLMQVVPQET) [28], which differs in only one amino acid from the mouse/rat sequence. On Western blots from the rat and mouse brain (see Additional file 1 for immunoblotting methods), the antibody recognized a single band corresponding to a molecular weight of approximately 46 kDa (Additional file 2), in agreement with a previous report [14]. LAT1 immunofluorescence resulted in intense staining of brain vessels and generally no parenchymal labeling, in accordance with previous immunostaining studies in mice and rats [14–18]. The high specificity of the immunostaining was further confirmed by identical LAT1 immunostaining and in situ hybridization patterns in specific regions such as the subcomissural organ, where intense LAT1 immunostaining was previously reported [15], and in the caudal part of the rat dorsal third ventricle, where we observed a very characteristic pattern of cells expressing high levels of LAT1 mRNA and protein surrounding the choroid plexus (see Additional file 2).

### Image analysis

Immunofluorescent images were captured using the following fluorescent filter sets: for Cy3, excitation of 540–590 nm, bandpass of 595 nm, and emission of 600–660 nm; for Alexa 488, excitation of 460–500 nm, bandpass of 505 nm, and emission of 510–560 nm. Semiquantitative image analysis was performed with ImageJ software (public domain at http://rsb.info.nih.gov/ij). In situ hybridization and immunofluorescent signals were compared between sections from a single experiment where all conditions were identical. Vascular LAT1 hybridization signal was quantified in the rat cortex where parenchymal hybridization signal is lower than in other forebrain regions, and thus, the more intense vascular signal could be easily distinguished. On darkfield images taken with a 5× objective, the intense vascular signal was separated from the low-level parenchymal signal using the threshold tool in ImageJ, and the area covered by the vascular signal was measured. LAT1 immunofluorescence in blood vessels was quantified on images taken with a 10× objective from the same regions of the mouse or rat cortex, hypothalamus and thalamus. Immunolabeled vessels were separated from the background using the threshold tool, and the average pixel intensity (brightness value) of the immunofluorescent signal was measured. Analyses from all brain regions yielded the same results, and data from the hypothalamus are presented.

### Statistical analysis

Data are presented as mean ± SEM. Groups were compared by the non-parametric Kruskal–Wallis test, and then post hoc comparisons between the LPS-treated groups *vs.* the control group were made by Dunn’s test.

## Results

### Distribution of LAT1 mRNA in the rat and mouse forebrain

**Fig. 1:**
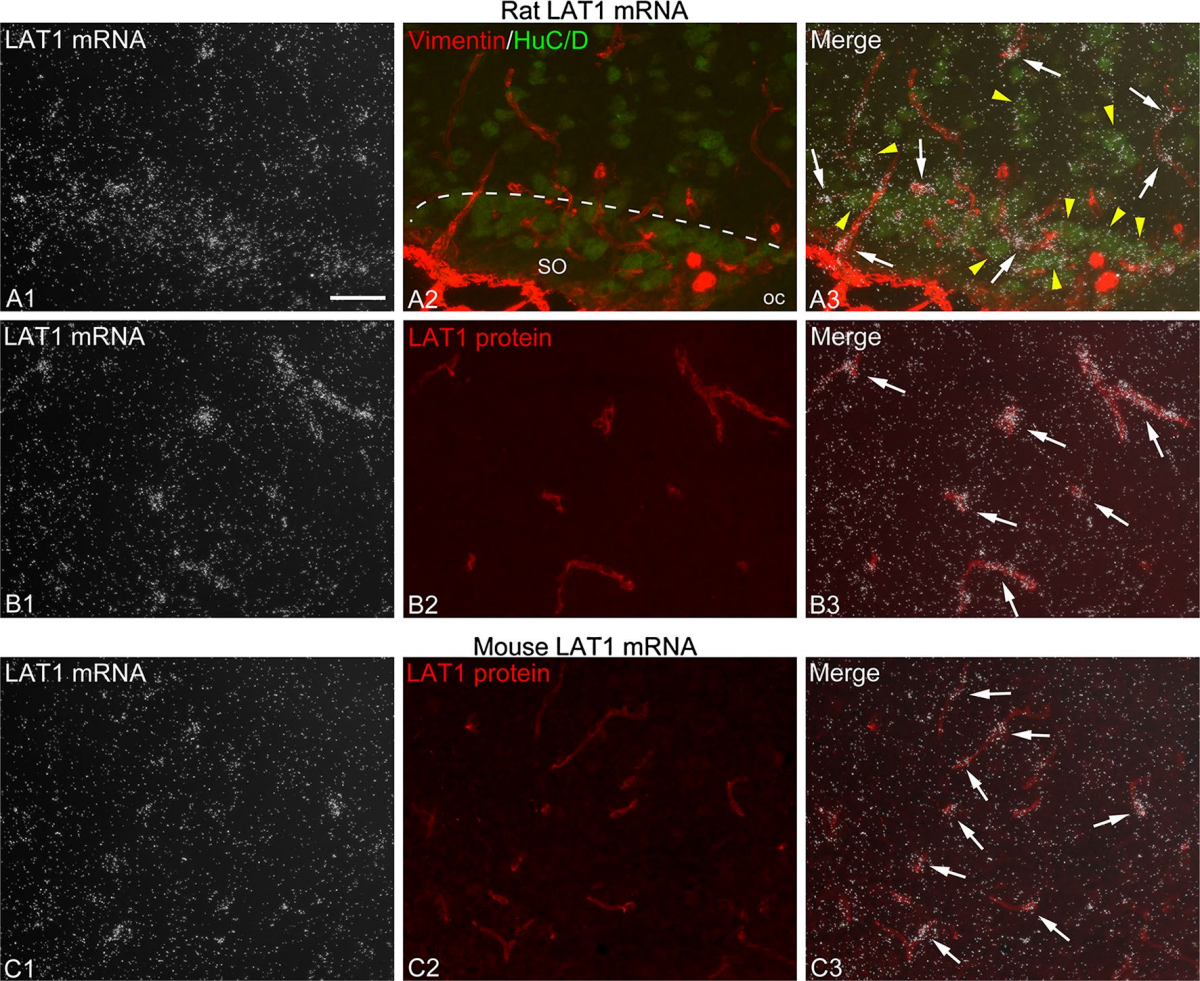
LAT1 mRNA is expressed in brain microvascular cells and neurons. **A** To confirm that LAT1 mRNA is expressed in multiple cell types, radioactive LAT1 in situ hybridization (**A1** darkfield image, silver grain accumulation represents LAT1 mRNA expression) was combined with dual immunofluorescence (**A2**) for vimentin (*red*) and HuC/D (*green*) to visualize the pericyte coverage of vessels and neuronal cell bodies, respectively. The area shown is from the rostral part of the rat hypothalamic supraoptic nucleus (SO). In the merge image (**A3**), note that silver grains form dense clusters over blood vessels (*arrows*), while a lesser degree of silver grain accumulation is present over neuronal cell bodies (*yellow arrowheads*). *oc* optic chiasm, *SO* supraoptic nucleus. **B**, **C** LAT1 in situ hybridization was co-localized with LAT1 immunofluorescence (*red*) that labels the vascular endothelium. Note that silver grain clusters that represent high LAT1 mRNA expression are formed over capillaries denoted by LAT1 immunofluorescence (*arrows* in **B3**, **C3**). Images are from the rat hypothalamic ventromedial nucleus (**B**) and the mouse hypothalamic arcuate nucleus (**C**). *Scale bar* 50 µm for all images

**Fig. 2:**
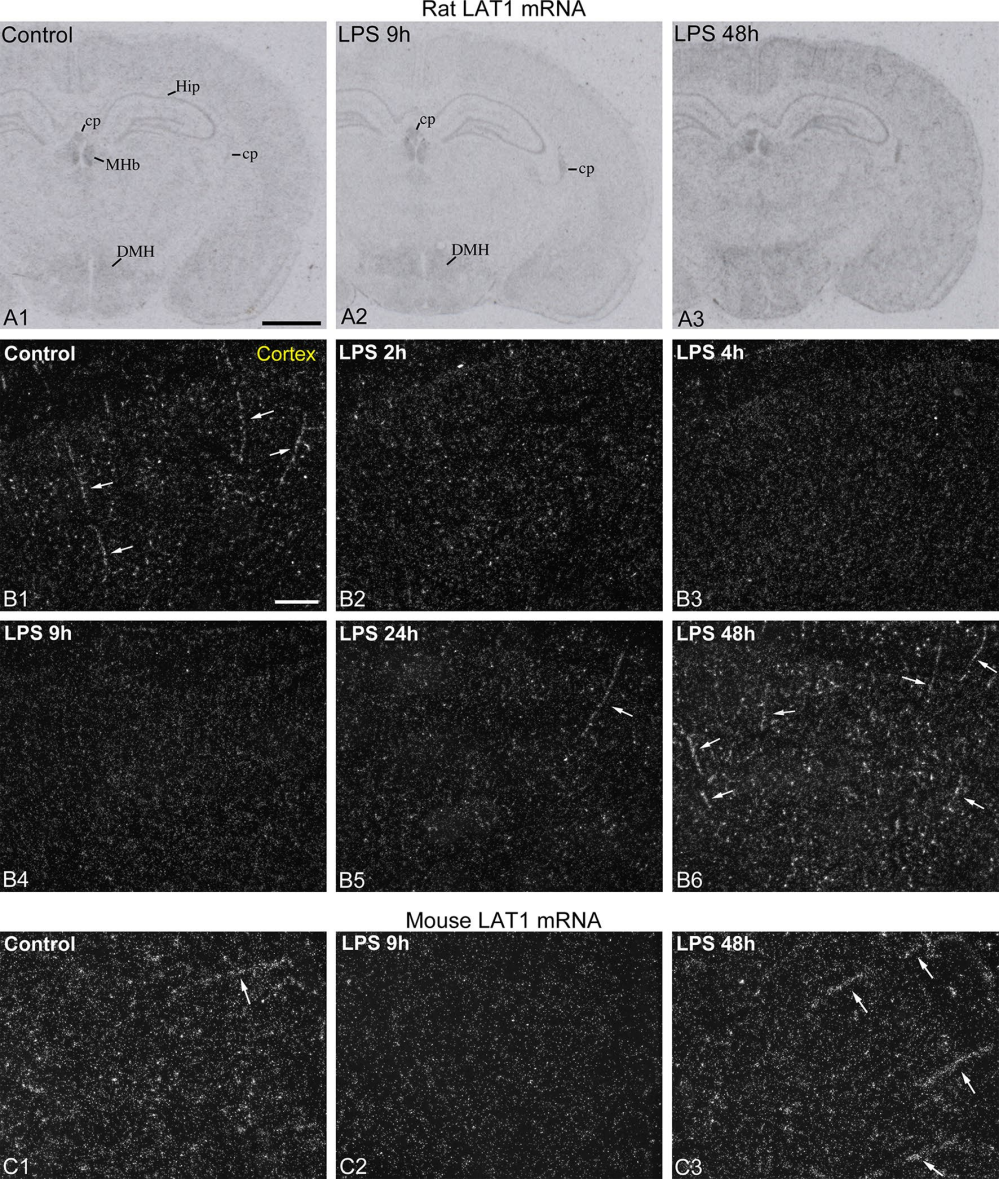
Effect of LPS on LAT1 mRNA expression in brain vessels of rats and mice. **A** X-ray film images of radioactive LAT1 in situ hybridization in rat forebrain sections. Note the reduced “graininess” of the labeling 9 h after LPS, which is especially visible in the thalamus. Neuronal labeling, well visible in the hippocampus and the hypothalamus, including the dorsomedial nucleus (DMH), is not altered. At 48 h after LPS, the “grainy” signal, which represents blood vessels, is more intense than in control brains over the entire section. *Cp* choroid plexus, *DMH* dorsomedial hypothalamic nucleus, *Hip* hippocampus, *MHb* medial habenular nucleus. *Scale bar* 2 mm. **B** Darkfield emulsion autoradiography images demonstrate the time course of LAT1 mRNA (silver grain accumulation, *white*) in the rat cortex. In the control cortex, intense hybridization signal is associated with major longitudinal vessels (*arrows*) and a multitude of small capillaries (numerous bright spots). This labeling vanishes by 4 and 9 h after LPS, only the much lower level parenchymal labeling (primarily neuronal) remains. At 48 h after LPS, hybridization signal labels a larger part of the vasculature than in controls, including major vessels (*arrows*) and capillaries. *Scale bar* 100 µm. **C** LAT1 mRNA in vessels of the mouse cortex. In the control brain, silver grains form clusters over major vessels (*arrow*) and capillaries, which disappear 9 h after LPS. 48 h after LPS, LAT1 hybridization signal in vessels is more intense than in controls. *Arrows* indicate major vessels. *Scale bar* 100 µm

LAT1 in situ hybridization patterns were highly similar in rats and mice. In general, intense hybridization signal was associated with blood vessels, including major vessels penetrating the brain surface and smaller vessels and capillaries (Figs. 1, 2B, C). This is in agreement with reported data that LAT1 mRNA is highly enriched in brain vessels [12, 18, 29, 30]. Parenchymal, non-vascular hybridization signal was generally less intense than the vascular signal, and only rarely resulted in the formation of well-defined silver grain clusters under the used experimental conditions. This parenchymal signal closely corresponded to the distribution pattern of different neuronal populations, and we confirmed that it was associated primarily with neuronal cell bodies (Fig. 1A); however, we cannot exclude that other cell types also contributed to the parenchymal signal. Neuronal LAT1 hybridization was more prominent in most hypothalamic nuclei, the medial habenular nucleus of the thalamus, certain amygdalar regions, hippocampal principal cell layers and pyriform cortex, whereas only light signal labeled most of the cortical areas and several thalamic nuclei (Fig. 2A). The choroid plexus was labeled with moderate hybridization signal in rats (Fig. 2A), while it was not labeled in mice, in agreement with previous mRNA studies [12, 14]. Interestingly, in the rat, cells with very intense hybridization signal were located alongside the choroid plexus of the dorsal third ventricle; caudally, these cells almost encircled the choroid plexus (see Additional file 2). Hybridization signal also labeled the subfornical and subcomissural organs (see Additional file 2), while hypothalamic tanycytes were not labeled.

### Effect of LPS on LAT1 mRNA

**Fig. 3:**
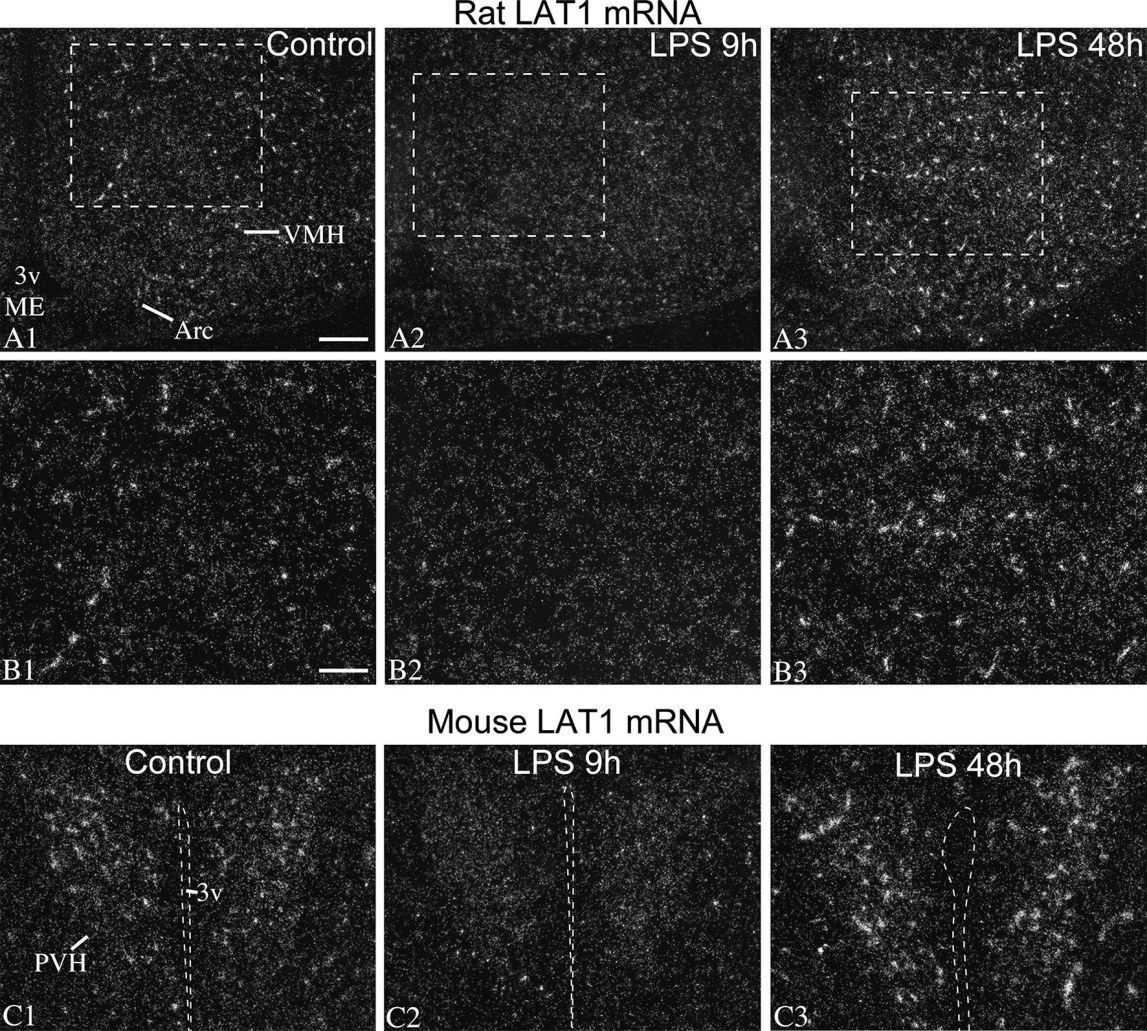
LAT1 mRNA decreased in vessels but not in neurons after LPS. **A**, **B** Darkfield emulsion autoradiography images show the distribution of LAT1 mRNA (silver grain accumulation, *white*) in the rat hypothalamus. *Boxed areas* from (**A**) are shown in higher magnification in (**B**). Low-level hybridization signal is distributed across the arcuate (Arc) and ventromedial nuclei (VMH), highly resembling a neuronal expression pattern; this signal does not change following LPS administration. Well-defined silver grains clusters over capillaries disappear 9 h after LPS, but become larger and more numerous at 48 h after LPS. *3v* third ventricle, *Arc*, arcuate nucleus, *ME* median eminence, *VMH* ventromedial hypothalamic nucleus. *Scale bar* 200 µm on **A**; 100 µm on **B**. **C** LAT1 in situ hybridization signal in the hypothalamic paraventricular nucleus (PVH) of mice. While LAT1 signal in capillaries virtually disappears 9 h after LPS, the low-level neuronal LAT1 signal in the paraventricular nucleus remains visible. At 48 h after LPS, hybridization signal in vessels is markedly increased. *3v* third ventricle, *PVH* hypothalamic paraventricular nucleus; *Scale bar* 100 µm

**Fig. 4:**
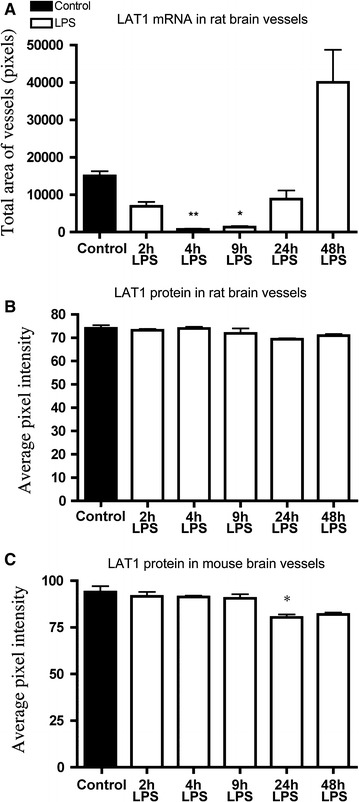
Semiquantitative image analysis results of LAT1 in situ hybridization and LAT1 immunofluorescence. **A** LAT1 hybridization signal was quantified in the rat cortex. Values represent area covered by vascular LAT1 hybridization signal. **B**, **C** LAT1 immunofluorescence was quantified in the rat and mouse hypothalamus, ventro-lateral to the hypothalamic paraventricular nucleus. Values represent the average brightness value of the immunofluorescence. Groups were compared by the non-parametric Kruskal–Wallis test and then by Dunn’s multiple comparison test. *P < 0.05; **P < 0.01; vs control; Samples sizes are n = 4 or 5 rats or mice/group

Following LPS administration, LAT1 mRNA decreased markedly in blood vessels of both rats and mice, in all regions of the forebrain. The time course of this response is illustrated in Figs. 2 and 3; quantification of this response by image analysis is presented in Fig. 4A. In rats, vascular hybridization signal decreased at 2 h after LPS. At this point, LAT1 mRNA was not detected in major vessels, only in smaller vessels and capillaries, but fewer than in controls (Fig. 2B). Hybridization signal in vessels could only occasionally be discerned at 4 and 9 h after LPS (Figs. 2B, 3A). At 24 h, the vascular labeling re-appeared but was still lower than in control rats (Fig. 2B). Forty-eight hours after LPS administration, the vascular signal was conspicuously and markedly higher than control levels in 3 out of 4 rats. In these rats, the hybridization signal was generally more intense in individual vessels and labeled a greater portion of the vasculature than in control rats (Figs. 2A, B, 3A). One rat in the 48 h group had signal levels comparable to the control group. This difference is probably due to the variation in the timing of the recovery phase after endotoxemia, and is reminiscent of the post-endotoxemic increase of OATP1c1 mRNA levels that could occur either 24 h or 48 h after LPS [10]. In mice, blood vessel-associated hybridization signal was only occasional or very light at 2 h and 24 h after LPS, and was completely absent in the 4 h and 9 h LPS groups (Figs. 2C, 3C). Similar to rats, in the 48 h LPS group, 3 mice had increased vascular LAT1 hybridization signal than controls, and 1 mouse had signal levels comparable to controls (Figs. 2C, 3C).

Parenchymal, neuronal LAT1 hybridization signal remained stable in forebrain regions at any time after LPS administration in both species (Figs. 2A, 3). LAT1 mRNA expression in the rat choroid plexus also did not change after LPS injection.

### Effect of LPS on LAT1 protein

**Fig. 5:**
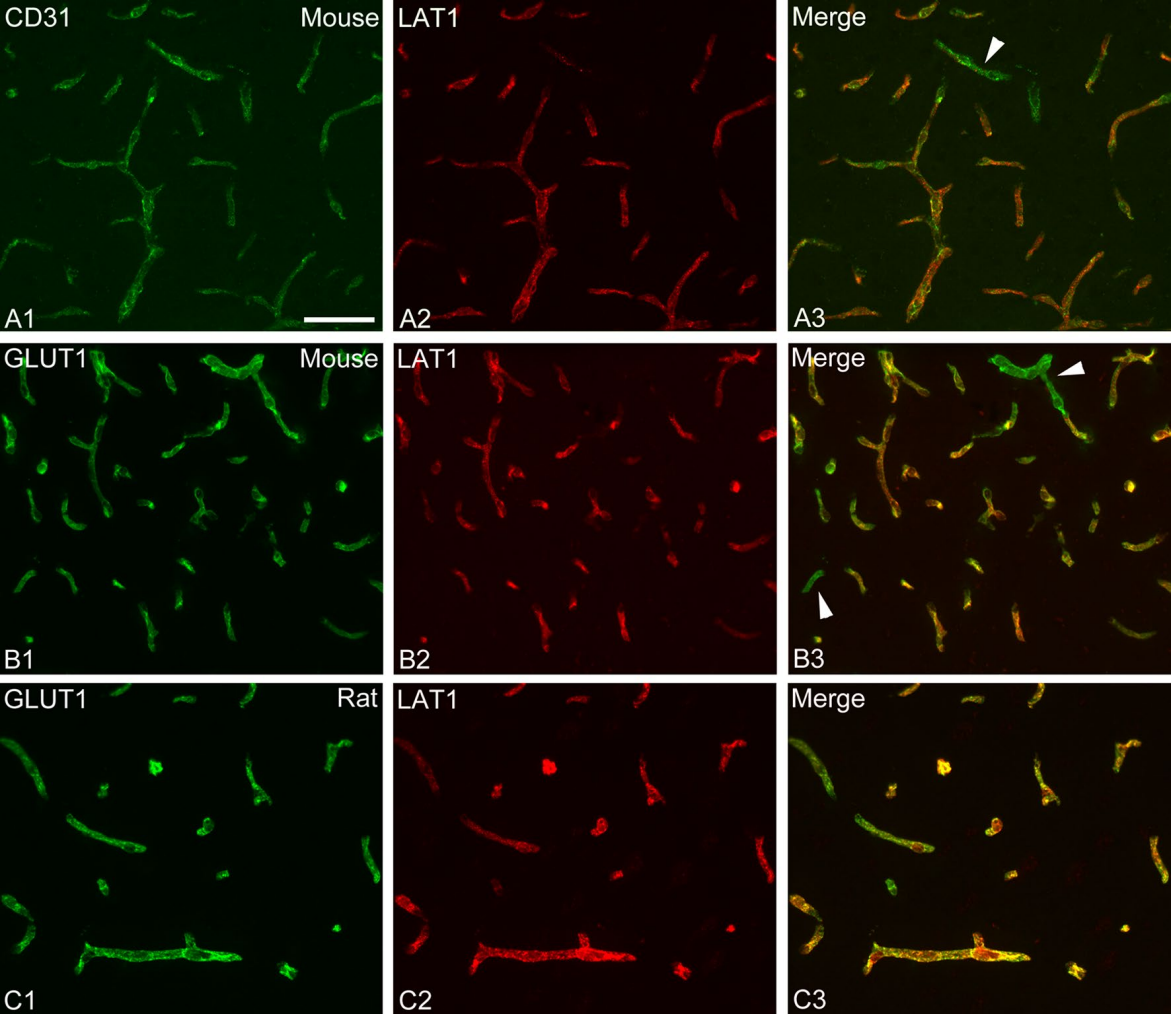
LAT1 protein is expressed in cerebral endothelial cells. **A1**–**A3** Dual immunofluorescence for the endothelial marker CD31 (*green*) and LAT1 (*red*) in the mouse brain. LAT1 always colocalized with CD31 in vessels. Some vessels that were labeled for CD31 apparently lacked or had only little LAT1 protein expression (*arrowhead*). Dual immunofluorescence for the endothelial marker GLUT1 (*green*) and LAT1 (*red*) and in the mouse (**B1**–**B3**) and the rat (**C1**–**C3**) brain. LAT1 always colocalized with GLUT1 in vessels. A fraction of vessels labeled for GLUT1 were not labeled for LAT1 (*arrowheads*). *Scale bar* 50 µm

**Fig. 6:**
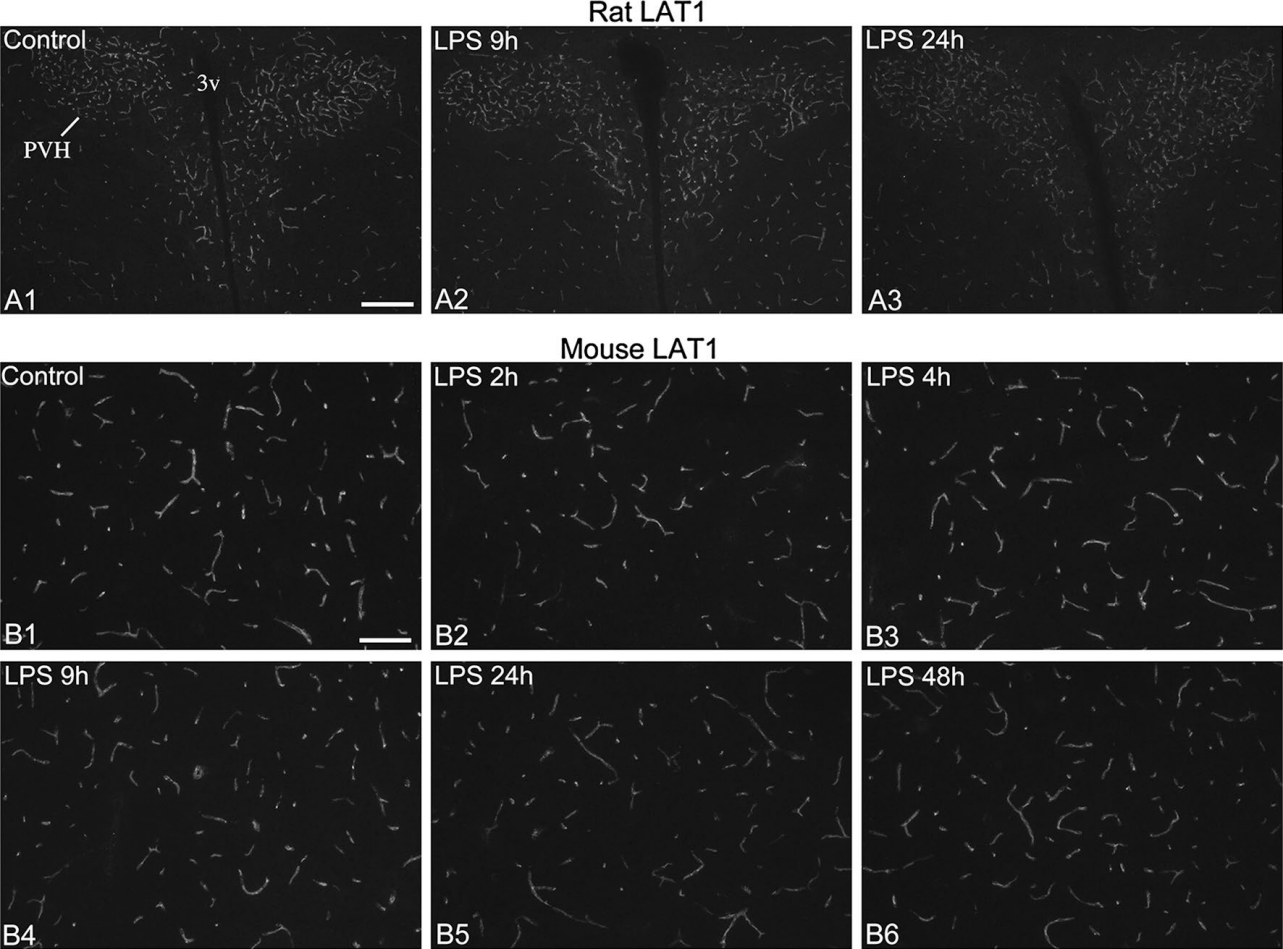
LAT1 immunofluorescence in brain vessels of the rat and mouse hypothalamus. **A** LAT1 immunofluorescence in the rat hypothalamus at the level of the paraventricular nucleus (PVH). The intensity of labeling tended to be lower at 24 h after LPS administration. *Scale bar* 200 µm. **B** LAT1 immunofluorescence in hypothalamic vessels of mice; labeling intensity is modestly reduced at 24 and 48 h after LPS. *Scale bar* 100 µm

LAT1 immunofluorescence labeled brain blood vessels in both species. Using dual immunofluorescence, we demonstrated that LAT1 in vessels always colocalized with the endothelial markers CD31 or GLUT1 (Fig. 5). In both rats and mice, the overall density of labeled vessels was essentially the same following LPS treatment as in controls. In rats, the signal intensity of vessels tended to be slightly lower in the 24 h LPS group, but was not significantly different from the control group (Figs. 4B, 6A). In mice, the brightness of LAT1 immunofluorescence in vessels decreased noticeably, albeit only modestly in the 24 h and 48 h LPS groups (Fig. 6B). By image analysis, the decrease in the 24 h group was statistically significant (Fig. 4C). This reduction of labeling intensity was observed in all brain regions, including the cortex, thalamus, hypothalamus and amygdala.

Interestingly, we did not observe clear LAT1 immunostaining in the rat choroid plexus, with the exception of a few occasional cells, mostly in the choroid plexus of the dorsal third ventricle. This stands in contrast to the presence of LAT1 mRNA, and a previous report that demonstrated LAT1 immunostaining in the epithelial cells of the rat lateral ventricle choroid plexus [18]. However, intense LAT1 immunostaining was present in the characteristic choroid plexus-associated cells that were labeled with intense LAT1 hybridization signal (see Additional file 2).

### Effect of LPS on LAT2 mRNA

**Fig. 7:**
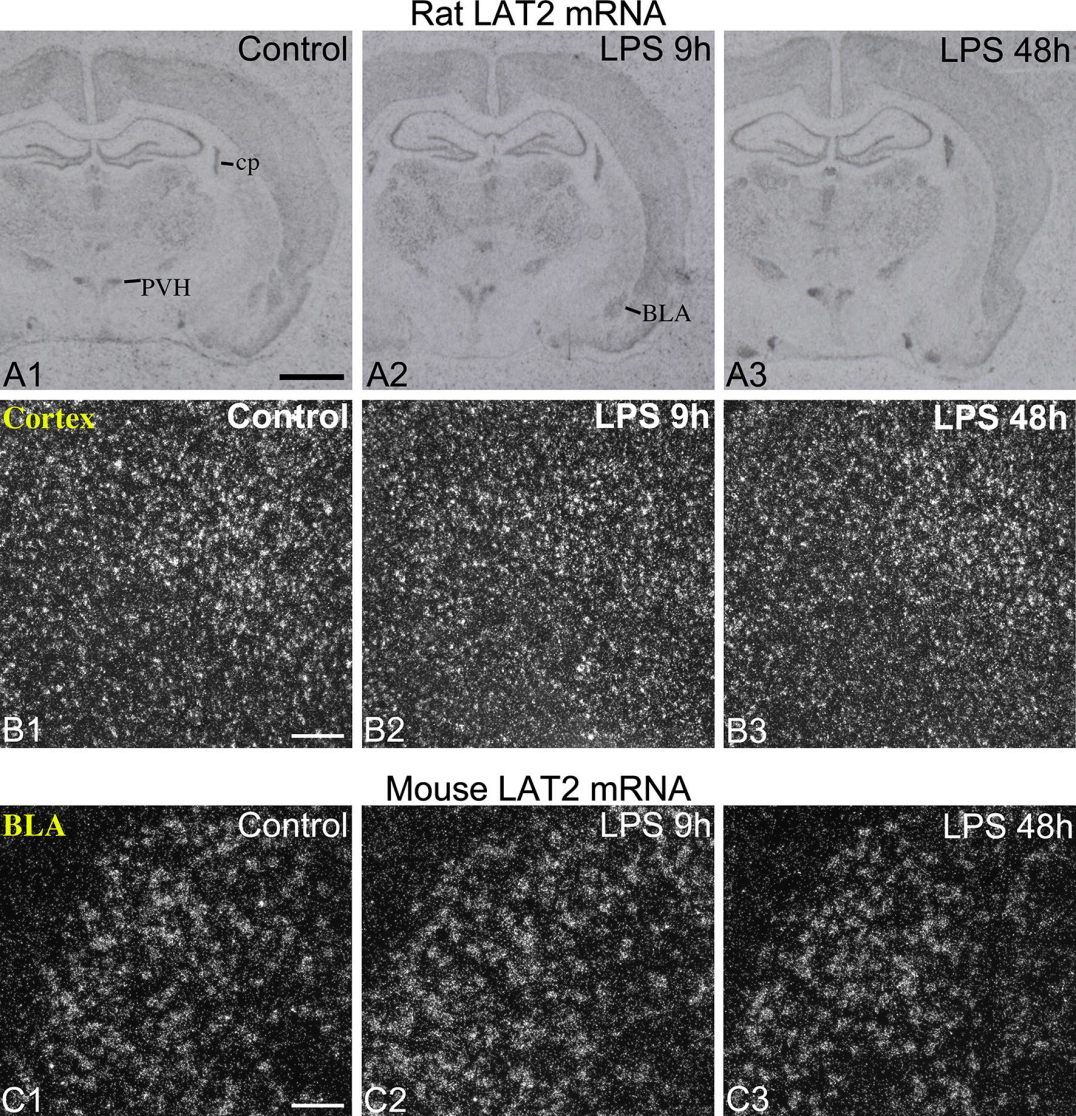
LPS had no effect on LAT2 mRNA expression in the rat and mouse brain. **A** X-ray film images of radioactive LAT2 in situ hybridization from the rat forebrain. LAT2 is expressed in several neuronal populations and the choroid plexus, but its expression did not change after LPS administration. *BLA* basolateral amygdaloid nucleus, *cp* choroid plexus, *PVH* hypothalamic paraventricular nucleus. *Scale bar* 2 mm. **B** Darkfield emulsion autoradiography images from the rat cortex show no change in neuronal LAT2 mRNA expression after LPS. *Scale bar* 200 µm. **C** Similarly, no change in LAT2 hybridization signal was observed in neurons of the basolateral amygdaloid nucleus in mice. *Scale bar* 100 µm

The pattern of LAT2 mRNA distribution was essentially the same in rats and mice, and corresponded to the in situ hybridization pattern described previously in mice [12, 19]. Intense neuronal expression was present in the cortex, hippocampus, amygdalar regions and numerous thalamic nuclei (Fig. 7A). In the hypothalamus, the paraventricular and supraoptic nuclei were intensely labeled (Fig. 7A), while most other nuclei had no or very low level of hybridization signal such as the arcuate and ventromedial nuclei. Moderate to intense expression was observed in the choroid plexus of both species (Fig. 7A). LPS did not affect LAT2 mRNA expression in either neurons or in the choroid plexus in either species (Fig. 7A–C).

Using LAT2 immunofluorescence, we did not observe any difference in the pattern or intensity of LAT2-immunoreactive labeling between control and LPS-treated mice and rats. However, the LAT2 antiserum is not completely specific to LAT2 in Western blot studies from the mouse brain [31], and we were unable to unambiguously confirm that the immunostaining patterns were entirely specific to LAT2.

## Discussion

In the present study, we report that systemic inflammation, induced by bacterial endotoxin, caused a robust decrease in LAT1 mRNA expression at the blood–brain barrier of rats and mice. This was followed by an apparent rebound effect with increased LAT1 mRNA levels during the recovery from endotoxemia. A modest decrease in LAT1 protein levels was also observed in mice at 24 h following the LPS stimulus. Neither LAT1 nor LAT2 mRNA levels were altered in the choroid plexus or in neurons following LPS administration.

With respect to TH homeostasis in the brain, perhaps the most important conclusion of the present study is that the downregulation of OATP1c1 and MCT8 at the blood–brain barrier are not accompanied by a compensatory increase in either LAT1 or LAT2 expression. The most remarkable aspect of the results, however, is that LPS administration causes strikingly similar changes in LAT1, OATP1c1 and MCT8 mRNA levels at the blood–brain barrier. The downregulation of LAT1 mRNA in brain vessels occurred essentially at the same time course and with the same robustness as that of OATP1c1 and MCT8 mRNAs [10]. Moreover, during the recovery phase from endotoxemia, LAT1 mRNA increased markedly in vessels, which is highly reminiscent to the rebound effect we observed for MCT8 and OATP1c1 mRNAs between 24 h and 48 h after LPS injection [10]. These parallel changes are somewhat surprising since LAT1 is a high affinity amino acid transporter that accounts for the uptake of large neutral amino acids into the brain [32, 33], and regarded only as a secondary TH transporter [11]. Thus, these data show that two different transport systems of the blood–brain barrier, one transporting primarily amino acids (LAT1) and the other primarily TH (MCT8 and OATP1c1), are regulated in a parallel manner in response to inflammation. This finding, therefore, raises the question as to whether there are other transporters at the blood–brain barrier that are similarly regulated in inflammatory conditions. Unfortunately, there are currently no reports in the literature about the in vivo effect of inflammation on major blood–brain barrier influx transporters. Interestingly, however, in a very similar LPS model to ours, organic anion transporter 3 and organic anion-transporting polypeptide 1a4 protein levels decrease in brain capillaries 24 h after LPS injection [34]. Organic anion transporter 3 is regarded as a brain-to-blood efflux transporter [35], while organic anion-transporting polypeptide 1a4 as both an efflux and influx transporter of anionic compounds [36]. These data further indicate that multiple different transporter systems are down-regulated at the blood–brain barrier during systemic inflammation. Future studies are required to elucidate the effect of inflammation on other blood–brain barrier transporters.

Given the parallel changes in LAT1, OATP1c1 and MCT8 mRNAs in brain vessels, it is reasonable to hypothesize that the same cellular mechanism may be behind the inflammation-induced down-regulation of these genes. Since the expression of these transporters remains stable in other brain cell types [10], this mechanism may be quite specific to brain endothelial cells. Interestingly, LAT1 expression is robustly induced in activated human T-cells via the nuclear factor kappa B pathway [37], whereas in our LPS model, LAT1 mRNA levels decrease in spite of nuclear factor kappa B activation in cerebral endothelial cells [38, 39]. These data indicate that regulation of LAT1 in brain endothelial cells is different than in other cell types of the body.

In contrast to the robust effect on LAT1 mRNA, we observed only a modest decrease in LAT1 protein levels in mouse brain vessels at 24 h after LPS injection. This varies from the robust reduction in OATP1c1 protein levels observed 24 h following LPS, but is similar to MCT8 protein that remains stable in vessels [10]. The time of delay between the decrease in mRNA and protein levels probably reflects the half-life of the protein, in this case suggesting a high stability for LAT1 protein in brain vessels. Indeed, a half-life of several days is not uncommon for plasma membrane proteins [40–47]. Therefore, it is conceivable that LAT1 protein in brain vessels is more robustly depleted during sustained inflammation caused by an actual bacterial infection, the course of which can take several days or weeks.

Future studies will also be necessary to determine whether LAT1 transport function in brain vessels decreases in short-term and/or prolonged inflammatory conditions. The rate of LAT1-mediated amino-acid transport across the blood–brain barrier depends on the number of functional LAT1 proteins in the luminal and abluminal surfaces of endothelial cell membranes [18]. LAT1-mediated transport is considered to be saturated at physiological serum amino acid concentrations [29, 48–50]. Therefore, fewer LAT1 proteins in endothelial membranes would likely result in reduced LAT1-mediated amino acid transport. It is possible that by the internalization of LAT1 from the membrane to intracellular pools, LAT1-mediated transport decreases while the amount of LAT1 protein remains stable. Determining the subcellular distribution of transporters in brain endothelial cells is challenging using conventional immunofluorescence techniques [18]. As a result, we could not determine whether the distribution of LAT1 shifts between cellular compartments. Nevertheless, our results raise the possibility that LAT1 transport function could decrease, even if only days after the onset of inflammation. Since both the amino acids substrates of LAT1 and TH are necessary for normal brain function, their reduced uptake could impede certain neural processes, perhaps contributing to reduced cognitive function, a general symptom of inflammatory infections. Whether reduced LAT1 transport is an adaptive response following the initial phase of inflammation and/or during the actual recovery phase, or maladaptive, remains to be understood.

## Conclusions

LPS-induced inflammation caused a robust decrease in LAT1 mRNA expression at the blood–brain barrier of rodents, and a modest decrease in LAT1 protein content. This finding, along with previous results on TH transporters, suggests that systemic inflammation simultaneously down-regulates the mRNA expression of different transport systems of the blood–brain barrier but the kinetics of changes at the level of transporter proteins are different. The results imply that the transport rates of various substances across the blood–brain barrier, such as amino acids and hormones, may be significantly altered during inflammation.

## Additional files

Additional file 1: Immunoblotting Methods. Additional file 2: Validation of the specificity of the LAT1 antiserum. (A) Western blot from the rat midbrain, mouse hypothalamus and mouse cortex; the LAT1 antiserum recognized a single band corresponding to a protein that migrates with the molecular weight of approximately 46 kDA. Loading control is glyceraldehyde 3-phosphate dehydrogenase (GAPDH). (B-D) LAT1 immunofluorescence and radioactive LAT1 in situ hybridization were performed in adjacent sections to show the high specificity of LAT1 immunostaining. (B, C) Both LAT1 immunofluorescence and in situ hybridization labeled the subcomissural organ (SCO) in both mice (B) and rats (C). (D) In rats, a group of cells with intense LAT1 immunofluorescent and in situ hybridization signals were located around the caudal portion of the choroid plexus of the dorsal third ventricle (d3v cp). Scale bar = 100 µm.

## Abbreviations

MCT8: monocarboxylate transporter 8
LAT1: l-type amino acid transporter 1
LAT2: l-type amino acid transporter 2
OATP1c1: organic anion-transporting polypeptide 1c1
TH: thyroid hormone
