# Supplementary material for: Endotoxin-induced inflammation down-regulates l-type amino acid transporter 1 (LAT1) expression at the blood–brain barrier of male rats and mice
Source: Fluids Barriers CNS. 2015 Sep 4;12:21. doi: 10.1186/s12987-015-0016-8 (PMC4559167; doi:10.1186/s12987-015-0016-8)
Supplement: Additional file 1: — Immunoblotting Methods. [file 12987_2015_16_MOESM1_ESM.pdf]

Immunoblotting: Tissue samples from the rat midbrain, mouse cortex and mouse hypothalamus were lysed in a RIPA lysis buffer (Cat# R0278, Sigma Aldrich) supplemented with 0.2 mM of a protease and phosphatase inhibitor cocktails I and II (Sigma-Aldrich, St. Louis, MO). A total of 40 µg of protein were resolved on 5-15% TGX precast gels (Bio-Rad, Cat# 456-1084) and transferred on PVDF membranes (Millipore). Following a blocking step, membranes were briefly washed and incubated overnight in the rabbit LAT1 antiserum (40ng/ml). Following washing in TBS-T milk, membranes were incubated for 1h in horseradish peroxidase-conjugated secondary antibody (Cat# NA9340, GE Healthcare Life Sciences). Membranes were thoroughly washed in TBS-T, and the immuno-detection was performed with ECL reagents (Amersham Biosciences) and exposure were recorded on HyBlot ECL film. To control for equal loading, membrane was stripped using a stripping solution (Thermo Fisher Scientific Inc., Cat# 21059) and reprobed with GAPDH antibody (Santa Cruz Biotechnology, Cat# 32233)
