# Supplementary figures and images for: Endotoxin-induced inflammation down-regulates l-type amino acid transporter 1 (LAT1) expression at the blood–brain barrier of male rats and mice
Source: Fluids Barriers CNS. 2015 Sep 4;12:21. doi: 10.1186/s12987-015-0016-8 (PMC4559167; doi:10.1186/s12987-015-0016-8)

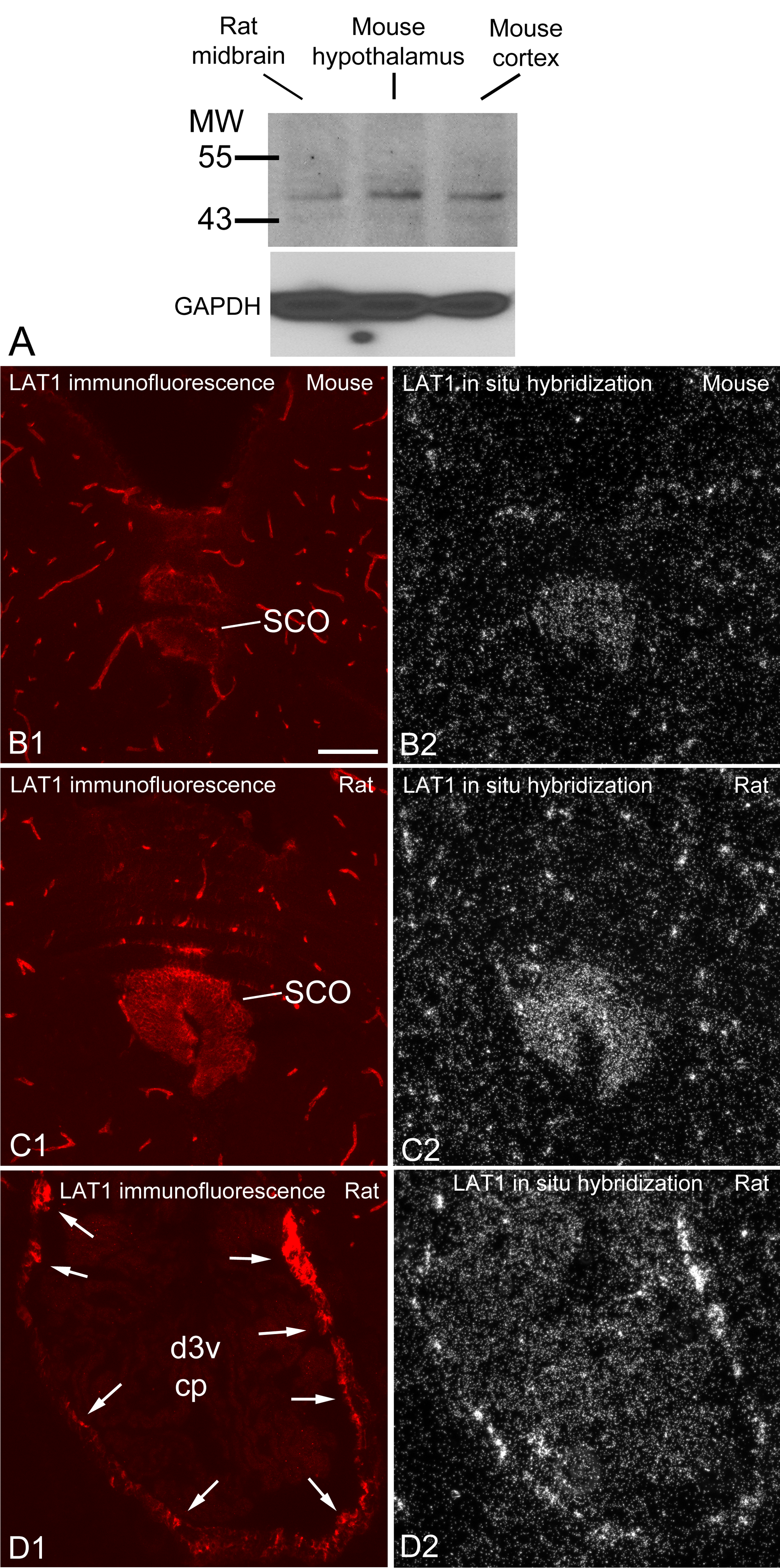

Supplement: Additional file 2: — Validation of the specificity of the LAT1 antiserum. (A) Western blot from the rat midbrain, mouse hypothalamus and mouse cortex; the LAT1 antiserum recognized a single band corresponding to a protein that migrates with the molecular weight of approximately 46 kDA. Loading control is glyceraldehyde 3-phosphate dehydrogenase (GAPDH). (B-D) LAT1 immunofluorescence and radioactive LAT1 in situ hybridization were performed in adjacent sections to show the high specificity of LAT1 immunostaining. (B, C) Both LAT1 immunofluorescence and in situ hybridization labeled the subcomissural organ (SCO) in both mice (B) and rats (C). (D) In rats, a group of cells with intense LAT1 immunofluorescent and in situ hybridization signals were located around the caudal portion of the choroid plexus of the dorsal third ventricle (d3v cp). Scale bar = 100 µm. [file 12987_2015_16_MOESM2_ESM.tif]
